# Similarities between pandemics and cancer in growth and risk models

**DOI:** 10.1038/s41598-020-79458-w

**Published:** 2021-01-11

**Authors:** Lode K. J. Vandamme, Ignace H. J. T. de Hingh, Jorge Fonseca, Paulo R. F. Rocha

**Affiliations:** 1 Faculty of Electrical Engineering, Eindhoven University of Technology, Eindhoven, The Netherlands; 2 Catharina Cancer Institute, Eindhoven, The Netherlands; 3 GROW-School for Oncology and Development Biology, Maastricht University, Maastricht, The Netherlands; 4 Urology Service, Champalimaud Foundation, 1400-038 Lisbon, Portugal; 5 Department of Electronic and Electrical Engineering, Centre for Biosensors, Bioelectronics and Biodevices (C3Bio), University of Bath, Claverton Down, Bath, BA2 7AY UK; 6 Department of Life Sciences, Centre for Functional Ecology (CFE), University of Coimbra, 3000-456 Coimbra, Portugal

**Keywords:** Cancer, Mathematics and computing, Health care

## Abstract

Cancer and pandemics are leading causes of death globally, with severe socioeconomic repercussions. To better understand these repercussions, we investigate similarities between pandemics and cancer and describe the limited growth in number of infections or cancer cells, using mathematical models. For a pandemic, the analysis shows that in most cases, the initial fast growth is followed by a slower decay in the recovery phase. The risk of infection increases due to the airborne virus contact crossing a risk-threshold. For cancers caused by carcinogens, the increasing risk with age and absorbed dose of toxins that cross a risk-threshold, may lead to the disease onset. The time scales are different for both causes of death: years for cancer development and days to weeks for contact with airborne viruses. Contamination by viruses is on a time scale of seconds or minutes. The risk-threshold to get ill and the number-threshold in cancer cells or viruses, may explain the large variability in the outcome. The number of infected persons per day is better represented in log–lin plots instead of the conventional lin–lin plots. Differences in therapies are discussed. Our mathematical investigation between cancer and pandemics reveals a multifactorial correlation between both fragilities and brings us one step closer to understand, timely predict and ultimately diminish the socioeconomic hurdle of both cancer and pandemics.

## Introduction

Humanity is currently confronted with an extraordinary challenge: the coronavirus disease (COVID-19), which is caused by severe acute respiratory syndrome coronavirus 2 (SARS-CoV-2). The socioeconomic impact emerging from pandemics, such as COVID-19, cholera and influenza, is astronomical, spanning from global scale quarantines and lockdowns, to intergovernmental disputes^1^.

In parallel, cancer remains a leading cause of death globally. According to the World Health Organization, it was responsible for nearly 10 million obits in 2018. This debilitating disease has a critical financial burden for both the patient and society. In the US alone, the total of all cancer-related healthcare costs, were more than $80 billion in 2015. In the European Union, healthcare spending of more than €57 billion was equally alarming^2,3^.

Early detection and prediction are critical in both these deadly challenges. Mathematical and physical models can provide a better understanding and timely flag complex realities, in addition to summarizing vital data for both governments and citizens, including patients. The aim of the current study is to demonstrate that in pandemics and cancer similar problems may be solved and timely flagged, using analogous mathematical and physical models. The growth in the number of cancer cells, viruses or infected people in pandemics may converge in a similar growth function. Cancer and pandemic problems can be diminished by realizing that for both diseases, the risk to get ill increases with exposure time either to toxins for cancer or to viruses for pandemics. Although the time scale is quite different, early diagnosis is in both cases crucial. The variability in the diseases is explained by the difference in individual number thresholds for cancer cells or viruses, below which the body can handle the problem. The individuality of each cancer makes the treatment difficult, because number thresholds can be quite different between individuals and cancer types. This also holds for the different growth rates of cancer cells. But the general picture remains.

There is a long history of models for exponential, limited and the sub-exponential growth^4–16^. Limited growth models can describe pandemics and growth in biology, botanic, and tumors. One reliable model often used to explain limited growth scenarios has been proposed by Verhulst^5,6^. Verhulst proposed a new rate equation for bacterial growth with a finite sugar stock. Verhulst coined his model as the logistic equation. In his model, the cumulative number, $N(t)$ of infected people over time t, gives an S-curve. The new cases per unit time (often one day) *vs* *t,* give rise to the pandemic curve. The important diagnostic tool, the pandemic curve, can be expressed as:

1 $$\begin{array}{c}dN(t)/dt\equiv \dot{N}\end{array}$$

Initially holds: $N\propto \dot{N}\propto {e}^{t/\tau }$. Afterwards, *N*(*t*) levels off to a value *M* and in the Verhulst-like model, $\dot{N}$ decreases as $\dot{N}\propto {e}^{-t/n\tau }$. There is a need to understand that real data show a slower recovery than in the growth: n > 1. We make a plea for log–lin plot of *N* and $\dot{N}$ instead of lin–lin plots, because the early warning exponential growth is presented as a straight line. Data between regions with different population size and number of infections can easily be compared in one plot and the noisy data is represented in a relative way.

With a simple physical model, we explain four behavior rules in pandemics: distancing, hand washing, mask and face shield and ventilation with moderate air flow. A trapping model is proposed to explain the effect of absorbed dose of airborne viruses. The absorbed dose is often denoted in literature as ‘dose × exposure time’ for the integral dose. The individual risk increases with age and the effect of absorbed toxin holds for developing cancer. For some cancers the *‘exposure* *time’* is unknown or ultimately doesn’t exist. In our work, the *‘exposure* *time’* accounts for contact with viruses provoking an epidemic or with toxins provoking cancer. We note that for cancer, the contact time, has a scale of years and a scale of seconds or minutes for airborne virus contamination.

We want to understand the variability in age to get ill and variability in gravity of the diseases. The proposed crossing of a risk-threshold (virus or aggressive malign and benign cancer cell multiplication) is not terminal but explains that a person becomes ill under different conditions. Yet, in this work, the crossing of a number-threshold means that growth is out of control and the disease can be deadly. Below the number-threshold, the body is in control and risk of dying is negligible.

## Exponential growth and (generalized) logistic growth

### The Poisson rate equation, exponential growth

$N(t)$ and $\dot{N}$ are given by:

2 $$\begin{array}{c}\frac{dN(t)}{dt}=\frac{N(t)}{\tau }\to N\left(t\right)=N\left(0\right){e}^{t/\tau }\end{array}$$

with $N\left(0\right)\equiv {N}_{0}=1$ is $\dot{N}$ for exponential growth:

3 $$\begin{array}{c}\dot{N}\stackrel{\scriptscriptstyle\mathrm{def}}{=}\frac{dN\left(t\right)}{dt}=\frac{{e}^{t/\tau }}{\tau }=\frac{N(t)}{\tau }\end{array}$$

The $\dot{N}$-curve is the pandemic curve describing the new infections per day. For cancer, the start of tumor growth and the ensemble averages are often exponential, as was recently observed in lung cancer^11,17^. The risk probability on cell growth or infection is given by:

4 $$\begin{array}{c}h\left(t\right)\equiv \frac{dN}{Ndt}=\frac{dlnN}{dt}=\frac{1}{\tau }\end{array}$$

Note, the risk is represented by the slope of the straight line in the ln *N* vs lin *t* curve and not the slope of the curve in lin–lin. A small *τ*-value is an important indicator for the gravity of a pandemic or cancer growth. The gravity of a pandemic is also expressed in the doubling time, *T*_*2*_ for *N* and $\dot{N}$. The doubling time of cancer cells may be 3 months, which means *τ* = 131 days. For a severe pandemic, T_2_ can be one day, which is *τ* = 1.4 days. Here, days, weeks, months and years are henceforth abbreviated as: d, w, m, y, respectively. The reproduction number, *R* is also used to qualify pandemics. It is defined as the ratio between $\dot{N}(t)$ [*d*^−1^] at $t+\Delta t$ and at *t*. Hence, *R* depends on the choice for $\Delta t$. Here, $\Delta t=1d$. Exponential growth means $\dot{N}\propto {e}^{t/\tau }$ and R > 1. In the recovery phase of a pandemic $\dot{N}\propto {e}^{-t/\tau }$ and *R* < 1. For the exponential growth, the relations between the parameters: *τ, R* and *T*_*2*_ are given by:

5 $$\begin{array}{c}R\stackrel{\scriptscriptstyle\mathrm{def}}{=}\frac{\dot{N}\left(t+\Delta t\right)}{\dot{N }\left(t\right)}=\frac{N\left(t+\Delta t\right)}{N\left(t\right)}={e}^{\Delta t/\tau }=1+\%\, change\to 2={\mathrm{e}}^{{T}_{2}/\tau }\end{array}$$

with $\frac{\Delta t}{\tau } < 1$, then the parameter *R* is defined as:

6 $$\begin{array}{c}R={e}^{1d/\tau }\cong 1+1d/\tau \end{array}$$

The parameter $\tau$ can be extracted, leading to:

7 $$\begin{array}{c}\frac{\tau }{1d}\cong \frac{1}{R-1}\end{array}$$

or, for *R* >  > 1 or $\frac{{\Delta {\text{t}}}}{{\uptau }} \ge {1}$:

8 $$\begin{array}{c}\tau =1d/lnR \to R={e}^{\left(1d\times ln2/{T}_{2}\right)}\leftrightarrow \% \,increase=\left(R-1\right)\times 100\%\end{array}$$

Equation (8) enables the calculation of parameter ${T}_{2}$ as:

9 $$\begin{array}{c}{T}_{2}=1d\times \left(ln2/lnR\right)=\tau \times ln2\end{array}$$

A daily increase by 39% in a pandemic means the reproduction number *R* = 1.39 and *τ* = 3 *d*. We note that small *τ*-values are an indication for the gravity of a dangerous epidemic, but large values aren’t. Table 1 is introduced to shows the strong difference in sensitivity between the parameters: *τ,* *T*_*2*_ and *R* for exponential growth. To be specific, a range in *R* between 1.01 and 1.4 results in a range in τ between 100 and 3 d.

**Table 1:** Parameters describing the exponential growth phase.

*R* reproduction number	% increase per day	*T*_*2*_ [*d*] doubling time	*τ* [*d*] characteristic time	1/*τ* [*d*^−1^] risk
1	0	∞	∞	0
1.023	2.3	30	43.3	0.023
1.104	10.4	7	10,1	0.099
1.21	21	3.63	5.24	0.191
2	100	1	1.44	0.694

The reproduction number R is close to one with a small range compared to the larger range in *τ* or *T*_*2*_. Therefore, the communication to a broad pubic on growth is better in doubling time, T_2_ or τ than in R-values. But R is also applicable for non-exponential growth and decay. The values T_2_ and *τ* characterize only exponential growth.

For a less steep and dramatic increase in N(t) than an exponential growth a sub-exponential growth is applied to study early outbreaks of infectious diseases^12–14^.

### The Verhulst model for limited growth

Verhulst proposed the logistic equation for bacterial growth with a finite sugar stock^5,6^. The logistic equation explains the often observed limited growth. The risk of growth in the logistic equation is the time dependency. Initially the risk is constant but after a certain time, t_inf_ it shrinks to zero. The time dependency was accomplished by multiplying *1/τ* in the Poisson equation by the factor, (1 − *N*(*t*)*/M*), with the sugar dependent parameter *M* > 0. Another study made by Richards, denoted this rate equation as the autocatalytic function and compared the three most used growth functions at that time: monomolecular, autocatalytic and Gompertz growth^4,10^. Up to date reviews of models can be found in literature^16,18,19^.

In a pandemic, the measured *τ* is in reality an effective value and depends among others on the virus intrinsic value of the incubation time but also on extrinsic contributions like contact frequency, exposure time and quality of contact tracing. For cancer, τ depends on the growth rate of typical cancer cells, but perhaps also on life style. The *M*-value depends on population size and group immunity. On the other hand, for cancer, *M* is the maximum number of tumor cells that can survive with the existing nutrients limited by the available supply by blood vessels and the presence of tumor killer cells. The *τ*-value depends on the division time of the tumor cell. The Verhulst rate equation and solution for $N(t)$ and $\dot{N}$ with *N*_*0*_ = 1 are given by^5,6^:

10 $$\begin{array}{c}\frac{dN(t)}{dt}=\frac{\left(1-\frac{N(t)}{M}\right)}{\tau }\times N\left(t\right)\to N\left(t\right)=\frac{M}{1+\left(M-1\right){e}^{- t/\tau }}=\frac{M{e}^{t/\tau }}{{e}^{t/\tau }+\left(M-1\right)} \to \dot{N}=\frac{M(M-1){e}^{-t/\tau }}{\tau {\left(1+(M-1){e}^{-t/\tau }\right)}^{2}}\end{array}$$

**Figure 1:**
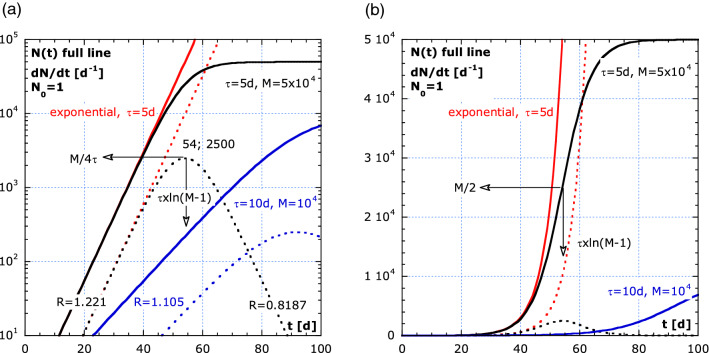
The exponential and limited growth in log–lin and lin–lin representation. (**a**) log–lin plot of the cumulative incidence, *N(t)* and the pandemic curve, the new infected persons per day: $\dot{N}\equiv \frac{dN}{dt}\cong \frac{\Delta N}{\Delta t}$ for $\Delta t< \tau$. The values: *R,* ${t}_{inf}$ and ${\dot{N}}_{max}$ depend on *τ* and *M*. (**b**) lin–lin plot of (**a**). The black line in (**b**) is the so-called S-curve, not visible in the log–lin plot. The small values are not visible to the human eye, if presented with large ones in a lin–lin plot.

**Figure 2:**
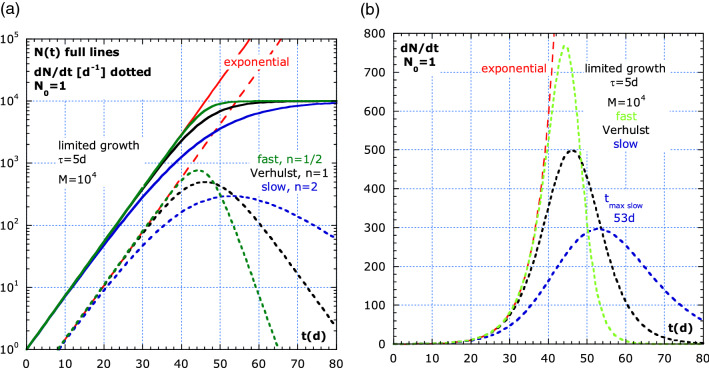
The Verhulst-like model in log–lin compared to a lin–lin representation of $\dot{N}(t)$. (**a**) The generalized logistic model or Verhulst-like model: $N\left(t\right)\, and \,\dot{N}(t)$ are compared between fast (n = ½), medium (n = 1) and slow (n = 2) recovery. The Verhulst-like solution with M → ∞ is nothing else than the exponential. (**b**) lin–lin of $\dot{N}$ in (**a**).

**Figure 3:**
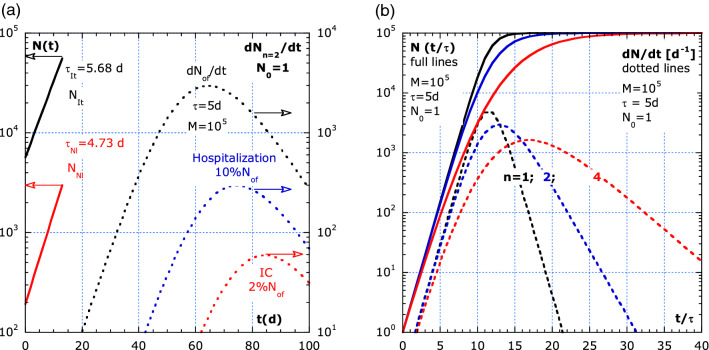
Merits of log–lin plots. (**a**) Real data (full lines) for the number of infections by COVID-19 pandemics in Italy (black) and The Netherlands (red) over only 13 days, *t* = 0 corresponds to 7 March 2020. The inferred *τ*_*Nl*_ = 4.7 d and *τ*_*It*_ = 5.7 d from the log–lin plot suggest a different handling by authorities. (**b**) The merits of a log–lin plot by the simulation of *N* (full lines) and $\dot{N}$ (dotted lines).

The cumulative growth, $N(t)$, results in an S-curve in a lin–lin plot, typical for sigmoid functions. On a log–lin scale, as in Fig. 1a, the exponential and limited growth starts as a straight line. At *t* = 0, we assume *N(0)* ≡ *N*_*0*_ = 1, which ascertains that growth starts with one infected person or one tumor cell for the simulations in Figs. 1, 2 and 3.

The $\dot{N}$-curve is used to monitor the pandemic. The limited growth is a pure exponential growth for $M\to \infty .$ The symmetric $\dot{N}$-curve starts as ${\dot{N}\propto e}^{t/\tau }$ and decays as ${\dot{N}\propto e}^{-t/\tau }$. Yet, we note that real data often shows non symmetric $\dot{N}$- curves, with a slower recovery than that of the growth phase. From the ‘1918-pandemic’ in Philadelphia^16^ we inferred from a log–lin plot the following:

11 $$\begin{array}{*{20}c} {\tau_{recovery} = 1.76 \times \tau_{growth} } \\ \end{array}$$

The Verhulst-like equation can fit these asymmetric $\dot{N}$-curves, as discussed in the following section of this manuscript.

In a virulent pandemic, in the absence of medicine and vaccines and at a time t^*^, we often observe stronger rules for quarantine. These rules are proposed to ‘flatten the pandemic curve’. One expects that stronger quarantine rules result in: *M*_*new*_ < *M* and *τ*_*new*_ > *τ.* With new quarantine circumstances, the infection process continues as depicted in Fig. 1a, but with new parameters. The new parameters are: ${1<N({t}^{*}=0)\equiv N}_{0}<{M}_{new}$ and *τ*_*new*_ > *τ*. By ignoring the incubation lag, the logistic equation comprises ${N}_{0}\ne 1$ as:

12 $$\begin{array}{c}N\left(t\right)=\frac{{N}_{0}\times {M}_{new}}{{N}_{0}+({M}_{new}-{N}_{0}){e}^{-t/{\tau }_{new}}}=\frac{{N}_{0}\times {M}_{new}\times {e}^{t/{\tau }_{new}}}{{N}_{0}\times {e}^{t/{\tau }_{new}}+({M}_{new}-{N}_{0})} \end{array}$$

In a similar trend, the number of cancer cells can also level off. The growth could be limited when the tumors need more nutrition and energy than the one currently available^20^. A change in *M* and *τ* can be provoked by a pharmaceutical agent to tackle metastasis. This results in irregular growth and is discussed in “Irregular growth kinetics for cancer and pandemics, simulation based on (12)” Section.

Figure 1a shows the *N(t)* as a function of time *t*, in a log–lin plot. The exponential and limited growth are represented by full lines. The simulation parameters are: *τ* = 5 d, *M* = 5 × 10^4^ and *τ* = 10 d, *M* = 10^4^. From the initially slope in the log–lin plot, an increase in number of patients by a factor of ten, in 12 days, is observed (*τ* = 5 d). The dotted lines show the symmetric $\dot{N}$-curves. The reproduction number for the exponential parts is $R={e}^{1/5}=1.221$ (black) and in the recovery phase is $R={e}^{-1/5}=0.818$. Around the top is $R\cong 1$ and beyond is 0 < *R* < 1.

The inflection point, for *N*_*0*_ = 1 on the *N(t)*-curve is indicated on the lin–lin version of Fig. 1a, in Fig. 1b. A higher *τ-* and lower *M*-value result in a lower maximum of the pandemic curve. A strong exponential growth shows a steep slope in the log–lin plot and a low *τ*-value. This is a warning to ‘flatten the $\dot{N}$-curve’, in order to keep the number of hospital patients below a critical level. The measures applied in behavioral epidemiology, to increase *τ* and decrease *M* span from home isolation, reducing contact frequency, controlling the size of contact bubbles, increasing social distancing, hygiene to timely tracing ‘super spreaders’, e.g., people that are not very ill but are spreading much more (10 to 100 times) viruses than contaminated people on the average.

### The asymmetric pandemic curve from the general logistic rate equation

Von Bertalanffy^8,9^ studied the growth rate in weight of animals. The time dependent growth was split in an anabolism term ($y$) *vs* weight ($W$) as $y\propto {W}^{\alpha }$ (where $\alpha$ is a dimensionless fitting exponent) and a catabolism term: $y\propto W$. In contrast to phenomenological models such as the logistic and generalized logistic ones, the refined mechanistic model by Von Bertalanffy, suggests an analysis in first principles. But this is at the expense of more unknown parameters and complex set of equations. Models as Susceptible-Infectious-Recovered (SIR) and Susceptible-Exposed-Infectious-Recovered (SEIR), have a set of 3 and 4 equations, respectively^16^. Therefore, the flexible Verhulst-like model is presented here.

The risk decreases with time, for a susceptible group to get infected. This is modeled by the coefficient $\left[1-{\left(N(t)/M\right)}^{1/n}\right]/\tau$ with $n>0$. For n = 1 holds the Verhulst-model. However, for $n\ne 1$, the convenient Verhulst-like rate equation results in an asymmetric $\dot{N}$-curve, starting as:

13 $$\begin{array}{c}\dot{N}\propto {e}^{t/\tau }\end{array}$$

and decaying as:

14 $$\begin{array}{c}\dot{N}\propto {e}^{-t/n\tau }\end{array}$$

with $n={\tau }_{decay}/{\tau }_{growth}$. The Verhulst-like equation starts as an exponential, which is in contrast to the sub-exponential growth. The rate equation is widely used and discussed^15^. The substitutions: ${\left(N(t)\right)}^{1/n}=T(t)$, $N\left(t\right)={\left(T(t)\right)}^{n}$ and ${M}^{1/n}=m$ change the Verhulst-like rate equation in the Verhulst-rate equation:

15 $$\begin{array}{c}\frac{dN(t)}{dt}=\frac{N(t)}{\tau }\left(1-{\left(\frac{N(t)}{M}\right)}^{1/n}\right)\to \frac{d{\left(T(t)\right)}^{n}}{dt}=n{T(t)}^{n-1}\times \frac{dT(t)}{dt}=\frac{{T(t)}^{n}}{\tau }\times \left(1-\frac{T(t)}{m}\right)\end{array}$$

Fast recovery occurs for 0 < n < 1. Slow recovery occurs for n > 1 and, for n = 1, the epidemic curve is symmetric. The solution for (15) in T(t) with the use of (10) is:

16 $$\begin{array}{c}\frac{dT(t)}{dt}=\frac{T(t)}{n\tau }\left(1-\left(\frac{T(t)}{m}\right)\right)\to T(t)=\frac{m}{1+\left(m-1\right){e}^{- t/n\tau }} , T\left(0\right)=N\left(0\right)=1\end{array}$$

with $$N(t)={\left(T(t)\right)}^{n}$$:

17 $$\begin{array}{c}N(t)=\frac{M}{{\left(1+({M}^{1/n}-1){e}^{-t/n\tau }\right)}^{n}} \end{array}$$

giving for $$N\left(0\right)={N}_{0}\ne 1$$:

18 $$\begin{array}{c}N\left(t\right)=\frac{M{N}_{0}}{{\left({{N}_{0}}^{1/n}+\left({M}^{1/n}-{{N}_{0}}^{1/n}\right){e}^{-t/n\tau }\right)}^{n}} \end{array}$$

The solution reported by Ma^16^, below his Eq. (8) with: *C(t)* = *N(t),* *K* = *M,* *r* = *1/τ* and *α* = *1/n* is wrong. Because the term $exp\left(-\frac{rt{K}^{\alpha }}{{K}^{\alpha }-{C}_{0}^{\alpha }}\right)$ in the 2nd Eq. on page 140 in the section, ‘Richards model’ should be: $exp\left(-r\alpha t\right)$, which is Eq. (18) in our symbols. The parameters: *n* and *M* are independent, hence, $n\ne 1-{M}^{-1/n}$ or in the notation used by Ma,^16^: $1/\alpha \ne 1-{K}^{-\alpha }$.

The Verhulst-like expressions for: *N(t),* $\dot{N}$, the maximum ${\dot{N}}_{max}$ at the inflexion time, *t*_*inf*_ and $N({t}_{inf})$ are summarized in Table 2. It shows the Eqs. (19–23) and (19c), (20c), for ‘slow’ decay with ${\tau }_{decay}=2{\tau }_{growth}$ and Eqs. (19a) and (20a) for ‘fast’ decay, with ${\tau }_{decay}={\tau }_{growth}/2$. The equations are used in the simulation of Fig. 2 to compare: ‘fast’, Verhulst-limited growth and ‘slow’ for $N$, $\dot{N}$, ${t}_{inf}$, ${\dot{N}}_{max}$ and $N({t}_{inf})$. The effect of the exponent *1/n* in the generalized limited growth model is outspoken in the $\dot{N}$- curves, in dotted lines, but have less effect in the *N(t)*-curves, in full lines.

**Table 2:** Verhulst-like equations with *N*_*0*_ = *1*, $n=\frac{{\tau }_{decay}}{{\tau }_{growth}}$ with *n* = ½, 1 and 2.

$N\left(t\right)=\frac{M{e}^{t/\tau }}{{\left[\left({M}^{1/n}-1\right)+{e}^{t/n\tau }\right]}^{n}}$ (19)		$\dot{N}=\frac{M\left({M}^{1/n}-1\right){e}^{-t/n\tau }}{{\tau \left[1+\left({M}^{1/n}-1\right){e}^{-t/n\tau }\right]}^{n+1}}$ (20)	
${t}_{inf}=n\tau \times ln\left[n\left({\left(M/{N}_{0}\right)}^{1/n}-1\right)\right]$ (21)		${\dot{N}}_{max}=\frac{M}{n\tau }\times \frac{1}{{\left(1+1/n\right)}^{n+1}}$ (22)	$N\left({t}_{inf}\right)=\frac{M}{{\left(1+1/n\right)}^{n}}$ (23)
	‘fast’ decay; n = 1/2 asymmetric	Verhulst; n = 1; ‘medium’ symmetric	‘slow’ decay; n = 2 asymmetric
$N\left(t\right)=$	$\frac{M{e}^{t/\tau }}{{\left[{(M}^{2}-1)+{e}^{2t/\tau }\right]}^{1/2}}$ (19a)	$\frac{M{e}^{t/\tau }}{\left(M-1\right)+{e}^{t/\tau }}$ (19b)	$\frac{M{e}^{t/\tau }}{{\left[\left(\sqrt{M}-1\right)+{e}^{t/2\tau }\right]}^{2}}$ (19c)
$\dot{N}=$	$\frac{M({M}^{2}-1){e}^{-2t/\tau }}{\tau {\left(1+({M}^{2}-1){e}^{-2t/\tau }\right)}^{3/2}}$ (20a)	$\frac{M(M-1){e}^{-t/\tau }}{\tau {\left(1+(M-1){e}^{-t/\tau }\right)}^{2}}$ (20b)	$\frac{M(\sqrt{M}-1){e}^{-t/2\tau }}{\tau {\left(1+(\sqrt{M}-1){e}^{-t/2\tau }\right)}^{3}}$ (20c)
${t}_{inf}$=	$\frac{\tau }{2}\times ln\left[\frac{{M}^{2}-1}{2}\right]$ (21a)	$\tau \times \mathrm{ln}\left(M-1\right)$ (21b)	$2\tau \times \mathrm{ln}\left(2(\sqrt{M}-1)\right)$ (21c)
${\dot{N}}_{max}=$	$\frac{2}{{3}^{3/2}}\times \frac{M}{\tau }$ (22a)	$0.25\times \frac{M}{\tau }$ (22b)	$\frac{4}{27}\times \frac{M}{\tau }$ (22c)
$N(t_{inf})=$	$\frac{M}{\sqrt{3}}$ (23a)	$\frac{M}{2}$ (23b)	$\frac{M}{2.25}$ (23c)

In Fig. 2, ‘fast’ (n = ½) shows for $\dot{N}(t)$ a higher maximum than ‘slow’ (n = 2; blue dotted line) for the same M-value. The log–lin plots show that the curves *N(t)* and $\dot{N}$ start proportional to ${e}^{t/\tau }$, from which *τ* can be calculated. Simulations with *n* > *1* fit better real pandemic outbreaks of influenza, Zika, Ebola^16^ and COVID-19^21^. Sub-exponential models only fit early growth. We infer from data on influenza, in 1918^16^, that *τ* = 3.6 d and *n* = 1.7.

A useful model fits evidences, with only a few parameters that supply information in a physical comprehensible manner. The reduction in risk for infection with n > 1 in Eq. (15), may be explained by an increase in path length and time between fewer susceptible persons and lesser spreaders of virus sources. An existing empirical power law capable of describing the number of infections per individual within a certain population size *P*, is given by:^22^

24 $$\begin{array}{c}y={y}_{0}{P}^{\beta }\to \frac{y}{P}={y}_{0}{P}^{\beta -1} \end{array}$$

with $\beta \ne 1$ in most cases. The indicator, *y* denotes the number of infections. For many urban indicators $\beta >1$. In biology, the value β = ¾ was often proposed wrongly as a universal constant. The pace of live decreases with increasing mass^23^. In biology a broad spectrum of phenomena scale over an immense range of mass with $\beta <1$ .

## The merits of log–lin plots: the slope of the tangent line in ln N(t) vs lin t is the risk

Testing the quality of noisy data and validating a hypothesis is easier from a log–lin plot, of $N\left(t\right)$ and $\dot{N}$, rather than from a lin–lin plot^24^. A straight line in a log–lin plot means an exponential growth, which means a constant relative change per time interval (percentage change).

Comparing countries in a pandemic, makes more sense in log–lin plots of $N\left(t\right)$ and $\dot{N}(t)$, rather than in lin–lin plots. Steep slopes are a warning. Inferring from a log–lin plot, the parameter *τ* is simple. The risk of infection for susceptible per number infected is given by^24^:

25 $$\begin{array}{c}h\left(t\right)\equiv \frac{dN}{Ndt}=\frac{dlnN}{dt}=1/{\tau }_{dyn}(t)\end{array}$$

The tangent line analysis in lin *N*(*t*) vs lin *t* gives *dN/dt*, not *h*(*t*). The change of the relative risk, on an event after a cancer treatment, was recently discussed with log–lin survival plots^24^. A log–lin plot is worth complicated statistical parameter testing.

The official diagnosed number of people, *N*_*of,*_ depends on the number of tests. *N*_*of*_ is often only 50% of the total number of infected people. The amount of virus patients in hospitals, *N*_*H*_, on intensive care, *N*_*IC*_*,* or deaths, *N*_*†*,_ are fractions of *N*_*of*_ (*t*)*.* For example, *N*_*of*_(*t*) = *f*_*1*_*N*(*t*)*;* *N*_*H*_(*t*) = *f*_*2*_*N*_*of*_(*t*)*;* *N*_*IC*_(*t*) = *f*_*3*_*N*_*of*_(*t*) and *N*_*†*_(*t*) = *f*_*4*_*N*_*of*_(*t*)*,* with, in general, not constant values for *f*_*4*_ < *f*_*3*_ < *f*_*2*_ < *f*_*1*_ < *1*. If the youngsters handle more viruses than the elderly people, then the fractions will depend on the parameter age. Data inhomogeneity often complicates the analysis.

Figure 3a shows real data from Italy and Netherlands (full lines). The simulation of three $\dot{N}$-curves (dotted line) with: *n* = 2, *M* = 10^5^, and *τ* = 5 d at the right hand scale, show the merits of log–lin plots. The effect of an arbitrarily chosen delay of 10 d between the rates: *N*_*of*_ (highest curve), *N*_*H*_ (10%), and *N*_*IC*_ (2%, lowest curve) results in parallel curves on log–lin scale. The low and high values are visible in log–lin plots and not in lin–lin plots. Figure 3b shows the merits of log–lin plots with a simulation for *N*(*t*) and $\dot{N}$ with Eqs. (10), (19c), (20c), *for* *n* = 1; *n* = 2; and (17) with *n* = 4.

## Risk increases with time, speculations on risk- and number thresholds that explain the variability in outcome

### Risk of infection increases with ‘exposure time’ for pandemics and for cancer

Airborne viruses are carried in small droplets and undergo Brownian motion. Brownian motion is at the origin of the diffusion time, till the virus is lodged at a sensitive spot in the body. The spread of the small droplets depends on the diffusion coefficient and is given by the diffusion length ${L}_{D}$:

26 $$\begin{array}{c}{L}_{D}=\sqrt{D\times t}\propto \sqrt{t} \end{array}$$

with *D* the diffusion coefficient of 0.25 cm^2^ s^−1^ for water vapor in air and *t* represent the time of diffusion. The virus concentration and the contact *‘exposure* *time’* are important. The effect depends on absorbed dose, as was often experimentally verified for toxins^25^. Large droplets with viruses go with the flow and fall on the ground. Larger droplets feel more the drag of the wind than the smaller ones that move more like Brownian motion. Large or small droplets depend on the speed of the air flow. In analogy, with the trapping model in physics, we propose that the risk of infection expressed as *1/τ* [s^−1^]*,* for a susceptible person in contact with a virus source, can be modeled as:

27 $$\begin{array}{c}1/\tau =\left(\phi vn\right) \end{array}$$

with *ϕ* the effective surface [cm^2^] of the receiver, or in a physicist language, the capture cross section. The capture cross section is reduced by a face shield and mask. The virus concentration *n* [cm^−3^] at distance *d* between virus source and receiver, is in a limited range $n\propto 1/{d}^{2}$. The concentration depends on the strength at the source, *n*_*s*_ and due to diffusion on the contact *‘exposure time’*. The proposed proportionality in a limited range of time and distance is:

28 $$\begin{array}{c}n\left(d\right)\propto {n}_{s}{\left(\frac{{L}_{D}}{d}\right)}^{2}\propto {n}_{s}\frac{Dt}{{d}^{2}}\to \frac{Dt}{{d}^{2}}<1 \end{array}$$

This is the physical basis of the distancing rule. The effect of *‘exposure time’* is important. The speed, *v* of airborne viruses travelling on large or small droplets depends either on the speed of the air flow or the thermal velocity of small droplets. The air quality is high if filtered and UV disinfected.

The risk reduction in a pandemic translates into an increase of *τ* and a reduction of *M*. Each cancer has its own risk factors and population at risk. In our work, the risk for the onset of tumor growth depends on predisposition (including cancer specific and population risks) and effects depending on time, viz*.* *‘stress-dose’* × *‘exposure time’*, and age. The absorbed dose has a mechanical (mesothelioma), chemical (toxins) or mental stress origin^25^.

### Speculation on variability in outcome of cancer and pandemics

Pandemics and cancer with small *τ*-values for growth are alarming. The risk, h(t), that cancer starts to develop, is assumed in analogy with the time dependent risk as^24^:

29 $$\begin{array}{c}h\left(t\right)=\frac{1}{{\tau }_{0}{(1+bt)}^{2}} \end{array}$$

${\mathrm{with}} \;b < 0\;{\mathrm{and}}\; 0 < t < -\frac{1}{b}$. The parameter *1/τ*_*0*_ [*y*^−1^] is the initial risk or predisposition for cancer. The parameter *b* [*y*^−1^] considers lifestyle, age (*bt*) and the absorbed dose impacts of stress, which may be a long list of pollutants, including benzene, pesticides, tobacco and alcohol; that disturbs the endocrine behavior. Long lasting inflammations or a lack of lymphocytes reduces the immune system. The role of some bacteria that induced cancer by e.g., genotoxic pks (+) *E. coli* must not be ignored^26^. These effects deteriorate the genes which control cell division.

**Figure 4:**
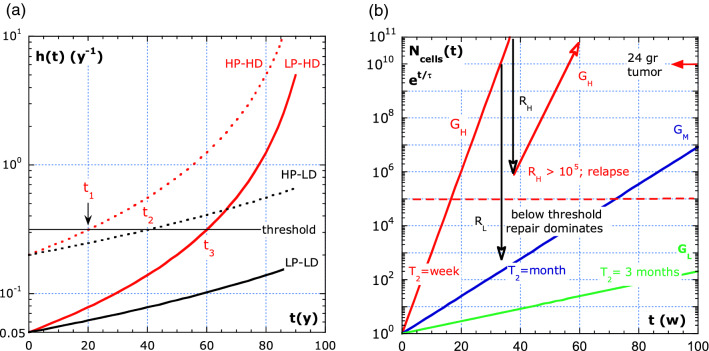
Exponential growth of cancer cells. (**a**) Variability in cancer due to an increase in risk with age of four individuals belonging to groups indicated by: H, high; L, low; P, predisposition; D, dose. The values for predisposition are: *τ*_*0*_ = 5 *y* (HP), *τ*_*0*_ = 20 *y* (LP). The effect of age and absorbed dose is reflected by the values: *b*= − 0.01 *y*^−1^ (HD), and *b* = − 0.005 *y*^−1^ (LD). (**b**) Exponential growth in cancer cells after crossing the risk-threshold illustrated in (**a**).

In Fig. 4a, above an arbitrary risk-threshold level of 0.314 [*y*^−1^], cancer starts to develop in a conceptual diagram. The crossing of the risk-threshold, at different ages, and cancer specificity explains the variability in outcome. The risk profile of four individuals is shown. The individuals belong to groups denoted as: High Predisposition and High Dose (HP-HD); High Predisposition and Low Dose (HP-LD); Low Predisposition and High Dose (LP-HD); and Low Predisposition and Low Dose (LP-LD). In our work, predisposition includes genetic susceptibility to cancer or infection, and simplistically accounts the different dynamics of the virus (and some cancers) in childhood and older adulthood. The LP-LD group, likely half of the world population, never reaches the risk-threshold in a lifetime. The risk-threshold is reached at the age, in years, of: *t*_*1*_ = 20 *y* for HP-HD; *t*_*2*_ = 40 *y* for HP-LD; and *t*_*3*_ = 60 *y* for the LP-HD group. The arbitrary chosen *τ*_*0*_ and *b*-values in Eq. 29 characterize the different groups. On the other hand, for an airborne virus infection, a risk of infection that increases with contact time may be similar to Eq. (28), inspired by the diffusion time, until the viruses are lodged (time scale in seconds or minutes).

The sketch in Fig. 4b shows the exponential growth of cancer cells. The start of a tumor growth is assumed at the risk-threshold times: *t*_*1*_*, t*_*2*_*,* or *t*_*3*_ in Fig. 4a. We note that our body contains about 6 × 10^13^ cells and a tumor of 242 gr counts about 10^11^ cells. A HeLa cell is about 2.42 ng^27^. In Fig. 4b, the number of cancer cells *N*_*cells*_ ranges from one to 10^11^. The number-threshold is arbitrarily chosen to be 10^5^ cells, for simplicity reasons equal for all cases. Below that number, the body stays under control. Above, the immune and repair system is out of control.

Surgery is one of the possible therapies for localized cancer, therefore in Fig. 4b the resection is indicated by vertical arrows (in black). Relapse occurs if the remaining cancer cells are above the number-threshold indicated by the red arrow. The cell growth rate, 1/*τ*, depends on the tumor type, with concomitant patterns of aggressiveness and growth. The arbitrarily chosen doubling times are: *T*_*2*_ = 1 w, 1 m and 3 m for the three types denoted as *G*_*H*_**,** *G*_*M*_ and *G*_*L*_. In Fig. 4b, the time scale is in weeks and the number-threshold is assumed the same for all. The variability in outcome may be predisposition, tumor type, absorbed dose effects and a different sensitivity resulting in different thresholds.

### Irregular growth kinetics for cancer and pandemics, simulation based on (12)

**Figure 5:**
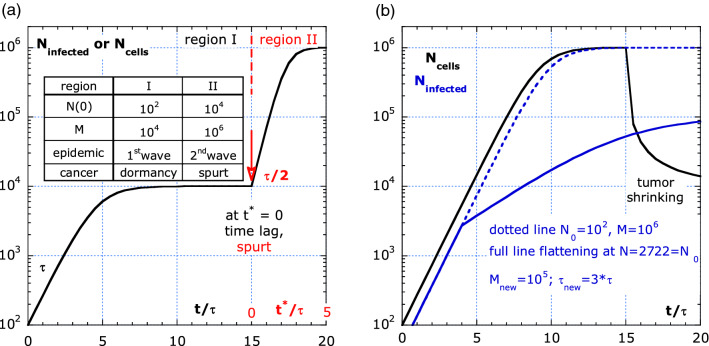
Irregular growth simulation with the Verhulst logistic equation, given by Eq. (12), with arbitrary parameters. (**a**) A sudden increase in risk results in a second wave in pandemics and a growth spurt in cancer. (**b**) A reduction of risk for growth can result in flattening the curve in pandemics or the tumor shrink by e.g., radiotherapy for cancer.

Growth plateaus were observed for breast cancer suggesting irregular growth kinetics^11,28^. Regularity is not a universal characteristic of malignant growth. Figure 5a,b, shows irregular growth, for cancer and pandemics respectively, with timescales normalized on the characteristic time *τ* at the start of each process. For the COVID-19 pandemics, *τ* is about 5 d and for cancer cell growth, *τ* is between 100 and 300 d. Figure 5a shows that after a period of dormancy the tumor can be triggered to disseminate very fast, the so-called growth spurt. Among others, angiogenesis^20^ and inflammations^28^ are two candidates at the origin of a growth spurt in cancer. A weakening of the behavior rules can trigger a second wave in a pandemics. The arbitrary parameters are indicated in the inset of Fig. 5a. Figure 5b shows the effect of a reduction in risk of infection by increasing *τ* by a factor of three and reducing *M* by a factor of ten. What happens without behavior measures is shown by the blue dotted line (*M* = 10^6^). The effect of cancer treatment is simulated by the black line.

## Conclusion

Our study reveals that the number of infections or cancer cells can be described using a limited growth mathematical model. Specifically, the mathematical model for limited growth proposed by Verhulst was used to explain the dynamics of both the on-going COVID-19 pandemics and cancer.

The analysis on Verhulst limited growth model reveals that:

- Early detection and prediction for both developing cancers and pandemics benefits from observations and analysis in a log lin format, where steep slopes are an important warning; In routinely used lin lin plots, small values are not perceptive to the human eye;
- The asymmetry in the growth and decay rate in a pandemic curve $\dot{N}\equiv dN/dt$ depends on $n={\tau }_{decay}/{\tau }_{growth}$. With n > 1, the N ˙-curve fits better the real data;
- The risk of developing cancer or an infection depends on different time scales. Years for toxin exposure in cancer and seconds or minutes for an infection by airborne viruses. The absorbed dose should be considered in the onset of an infection and cancer;
- Behavioral rules in a pandemic including confinement, social distancing, masks and face shields and air conditioning can be explained using physical models such as the trapping model and Brownian motion. The analogy reveals that apart from a few early diagnostic tests in cancer, an early detection of a pandemic is easier than that of cancer.

Ultimately, our multifactorial analysis contributes to timely diagnose cancer and early predict pandemic peaks.
